# Anticoagulation for Splanchnic Vein Thrombosis in Moderate-Severe Acute Pancreatitis: In-Hospital Outcomes from a Prospective Cohort Study

**DOI:** 10.3390/jcm15207824

**Published:** 2026-10-10

**Authors:** Lan Li, Xiaowen Mou, Qingyuan Tan, Yun Ma, Lihui Deng, Ziqi Lin, Tao Jin, Qing Xia

**Affiliations:** 1 West China Center of Excellence for Pancreatitis, Institute of Integrated Traditional Chinese and Western Medicine, West China Hospital, Sichuan University, Chengdu 610041, China; lilan@wchscu.cn (L.L.);; 2 West China Biobank, West China Hospital, Sichuan University, Chengdu 610041, China

**Keywords:** acute pancreatitis, splanchnic vein thrombosis, anticoagulation, in-hospital outcomes, bleeding risk

## Abstract

**Background**: Splanchnic vein thrombosis (SVT) is a common complication of acute pancreatitis (AP), but the role of anticoagulation (AC) during acute hospitalization remains unclear. We aimed to evaluate the impact of in-hospital AC on SVT recanalization and bleeding risk. **Methods**: We conducted a single-center prospective cohort study (2023–2026) including patients with moderate-severe AP and CT-confirmed SVT. The primary outcome was SVT recanalization at discharge; secondary outcome was in-hospital bleeding complications. **Results**: Among the 258 included patients, 89 (34.5%) received AC. The median AC duration was 42 days (range 1–83). SVT recanalization did not differ between AC and non-AC groups (15.7% vs. 16.6%, *p* = 0.862). Bleeding risk was significantly higher with AC (27.0% vs. 9.5%, *p* < 0.001), driven predominantly by clinically significant hemoglobin decline (7.9% vs. 1.2%, *p* = 0.015), while major bleeding did not differ significantly between groups. Among the anticoagulated patients, those with recanalization had a higher rate of necrosectomy (78.6% vs. 53.3%, *p* = 0.080). **Conclusions**: In this observational cohort, in-hospital AC was not associated with improved short-term SVT recanalization but was associated with a higher incidence of bleeding complications, driven predominantly by clinically significant hemoglobin decline. These observations require confirmation in randomized trials.

## 1. Introduction

Acute pancreatitis (AP) is a common inflammatory disorder in which approximately 30% of patients develop local or systemic complications, including splanchnic vein thrombosis (SVT) [1]. The reported incidence of SVT ranges from 13% to 17% [2,3] but exceeds 30% in patients with necrotizing disease [4]. The splenic vein is the most frequently involved, followed by the portal and superior mesenteric veins [5]. SVT can lead to serious sequelae, including portal hypertension, gastroesophageal varices, mesenteric ischemia, and life-threatening gastrointestinal bleeding [6].

Anticoagulation (AC) would seem indicated to prevent thrombosis progression, achieve recanalization, and prevent recurrent SVT. Supporting this, Zhou et al. found that early AC reduced SVT incidence (21.3% vs. 46.2%) and mortality [7]; Pagliari et al. and Chandan et al. reported significant recanalization improvement [8,9]. However, patients with AP are at significant risk of bleeding from gastroesophageal varices or pseudoaneurysms, making AC potentially catastrophic and the risk–benefit balance unclear [6].

Despite this uncertainty, current guidelines remain silent on this issue [10], and existing meta-analyses have reported conflicting results [11,12]. Clinical practice also reflects this uncertainty: an international survey found that only 44% of pancreatologists routinely prescribe therapeutic AC for SVT, with bleeding risk cited as the main concern by 77% of respondents [13]. To better characterize the existing evidence, we systematically summarized 13 studies, including 6 retrospective cohort studies, 1 prospective study, and 6 meta-analyses (Table 1). Reported recanalization rates varied widely across studies, with AC groups ranging from 18% to 69% and non-AC groups from 27% to 47%, and bleeding rates from 0% to 50% with AC vs. 0% to 15% without. Overall, the existing evidence is predominantly retrospective and conflicting, highlighting a critical need for prospective data. Therefore, we conducted a prospective cohort study to evaluate the impact of AC on recanalization and bleeding risk in AP-associated SVT.

## 2. Materials and Methods

### 2.1. Study Design and Setting

This prospective cohort study was conducted at West China Hospital, the largest AP center in China. The study protocol was approved by the Biomedical Research Ethics Committee of West China Hospital, Sichuan University (Approval No. 837; approval date: 31 May 2022). Informed consent was obtained. Given the absence of definitive guideline recommendations and the reliance on expert consensus for AC decisions, the study was designed as an observational cohort reflecting real-world clinical practice. This was an exploratory prospective cohort study in which all consecutive eligible patients with CT-confirmed SVT were enrolled during the study period (April 2023 to May 2026). No formal sample size calculation was performed prior to the study, as the study was not designed to test a pre-specified effect size; the sample size was determined by the number of consecutive eligible patients enrolled during the study period.

### 2.2. Inclusion and Exclusion Criteria

AP was diagnosed by two out of three criteria: epigastric pain, amylase/lipase > 3× upper limit of normal, or characteristic imaging. Moderately severe AP was defined as the presence of transient organ failure (resolving within 48 h) and/or local or systemic complications without persistent organ failure; severe AP was defined as persistent organ failure (>48 h) involving one or more organ systems [24]. SVT was diagnosed on the first contrast-enhanced CT (CECT) after admission, defined as venous thrombosis, intraluminal filling defects, vascular occlusion, luminal narrowing, or collateral vessel formation; imaging from referring hospitals was reviewed when required. Routine screening for venous thromboembolism (i.e., deep vein thrombosis or pulmonary embolism) was not performed; evaluation was only carried out when clinically indicated. Confirmed AP patients with SVT were included regardless of disease episode number, concomitant venous thromboembolism, or prior pancreas-related surgery. Exclusion criteria included chronic pancreatitis, pancreatic cancer or other malignancies, and mild acute pancreatitis.

### 2.3. Computed Tomography Examination

CECT scan of the abdomen and pelvis were assessed by two experienced radiologists blinded to clinical outcomes as part of a pancreatitis multidisciplinary team (MDT). Inter-observer discrepancies were resolved by consensus; when consensus could not be reached, a third senior radiologist adjudicated the final interpretation. A follow-up CECT scan was performed before discharge (or earlier if clinically indicated) to assess thrombus resolution or extension. In patients with persistent thrombus, scans were repeated at 1–3 month intervals until resolution.

### 2.4. Therapeutic Anticoagulation Treatment

In the absence of clinical guidelines, therapeutic AC decisions were made by the MDT based on individual patient assessment, and AC was initiated after MDT assessment following SVT diagnosis; the exact timing for each patient is recorded. Consistent with a recent DELPHI consensus [25], AC was typically given for portal or superior mesenteric vein thrombosis, or splenic vein thrombosis with evidence of progression (e.g., increasing luminal narrowing or filling), with or without extension into the superior mesenteric vein, while a watch-and-wait policy was adopted for non-propagating isolated splenic vein thrombosis [17,26]. AC was defined as therapeutic-dose anticoagulation. AC regimens included low molecular weight heparin (LMWH), warfarin, or direct oral anticoagulants (DOACs). The type, dosage, and duration of AC were recorded.

### 2.5. Outcomes

The primary outcome was SVT recanalization assessed on the last CECT before discharge, defined as re-established blood flow without narrowing, stenosis, or collateral formation. Discharge was chosen as the assessment point because it represents the end of in-hospital management and the earliest clinically relevant time at which a treatment decision must be made. The interval from SVT diagnosis to follow-up CECT was recorded for each patient. Secondary outcomes included bleeding complications classified according to ISTH criteria [27]: major bleeding; clinically relevant non-major bleeding (CRNMB; intra-abdominal, upper/lower gastrointestinal hemorrhage, or clinically significant hemoglobin decline); and minor bleeding (positive occult blood in urine or feces).

### 2.6. Quality Assurance

Data were collected on thrombosis location, thrombosis-related complications (gastroesophageal varices, collateral formation, splenic infarction, portal cavernoma, portal hypertension, ascites, liver failure, small bowel ischemia and necrosis), recanalization, and bleeding complications. Quality assurance, quality control, standard operating procedures, and researcher training were implemented. Data were collected by experienced clinical researchers (LL and XM) using a pre-defined pro forma designed by senior authors (ZL and TJ) and audited by clinical leads (LD, TJ, ZL, and QX).

### 2.7. Statistical Analysis

Categorical data are expressed as numbers and percentages and compared by *χ*^2^ test or Fisher’s exact test. Continuous data are presented as median and interquartile range (IQR) and compared using the Mann–Whitney *U* test if distribution was skewed. Multivariable logistic regression was performed. The baseline variables that differed significantly between groups were modified computed tomography severity index (mCTSI), acute necrotic collections, and necrosis involving the pancreatic body and tail. Because mCTSI incorporates acute necrotic collections and the extent of pancreatic necrosis, it captures these three imbalances within a single composite score. To avoid overfitting given the sample size, the model was therefore restricted to mCTSI as a single integrated covariate. A parsimonious model was used, and the results of multivariable and subgroup analyses should be interpreted as exploratory. Results are presented as adjusted odds ratios (ORs) with 95% confidence intervals (CIs). A two-sided *p* < 0.05 was considered statistically significant. Statistical analyses were performed using SPSS^®^ 21.0 (IBM, Armonk, NY, USA).

## 3. Results

### 3.1. Patient Characteristics

**Figure 1:**
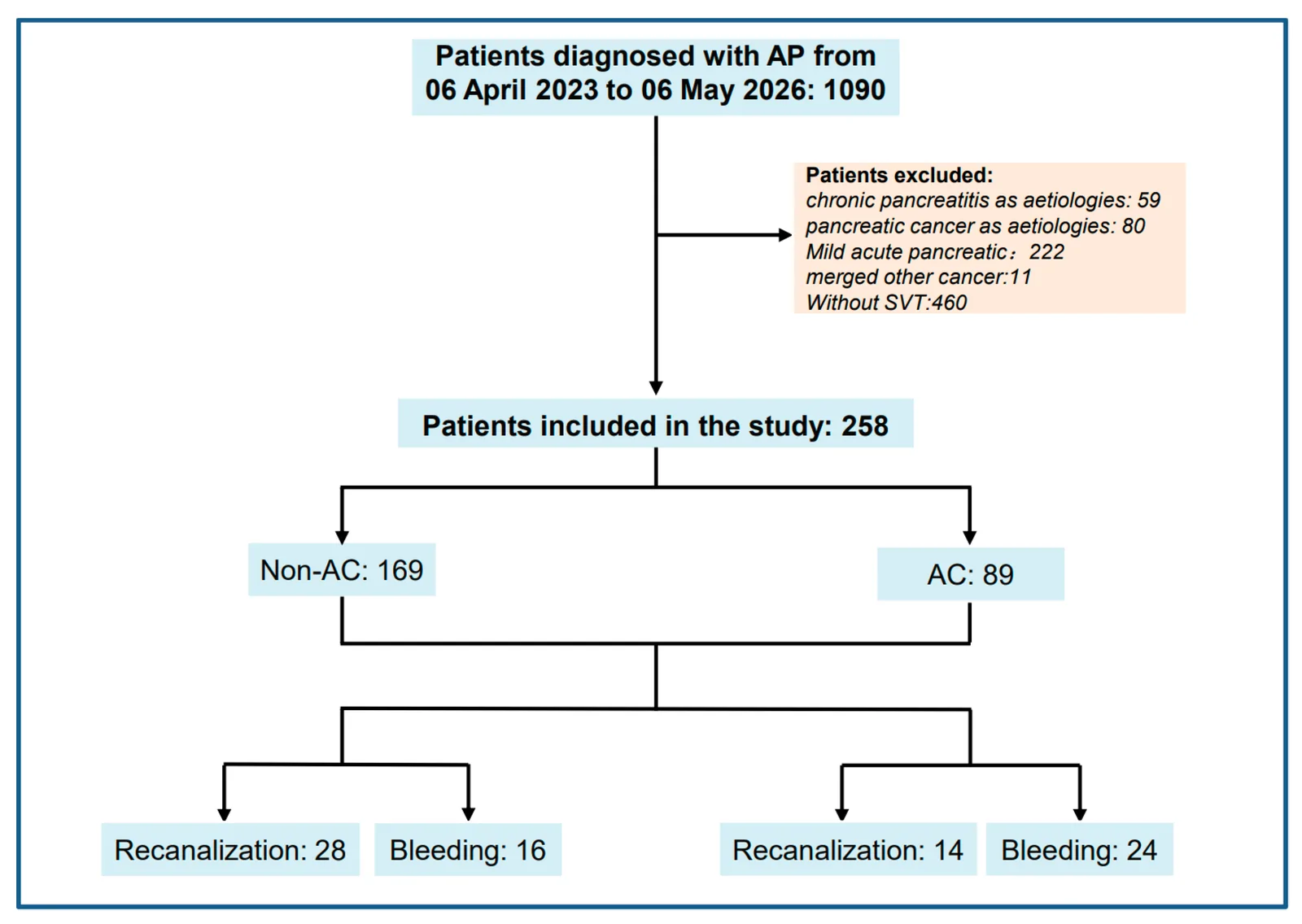
Patient selection process of acute pancreatitis and splanchnic vein thrombosis. AP, acute pancreatitis; SVT, splanchnic vein thrombosis; Non-AC: without therapeutic anticoagulation; AC: anticoagulation.

Of the 1090 AP patients screened, 258 met the inclusion criteria after excluding 59 with chronic pancreatitis, 91 with pancreatic cancer or other malignancies, and 682 with mild severity or no SVT on CT, yielding an overall SVT incidence of 23.7% (Figure 1). Baseline characteristics are shown in Table 2. Median age was 47 years (range 24–77), 68.6% were male, and hypertriglyceridemia was the leading etiology (46.1%). Local pancreatic complications were common: 76% had acute peripancreatic fluid collections, 38.4% had acute necrotic collections, and 51.9% underwent pancreatic necrosectomy.

The AC and non-AC groups were generally well-balanced at the baseline, with the exception that the AC group had higher mCTSI (*p* = 0.023), higher rates of acute necrotic collections (47.2% vs. 33.7%, *p* = 0.035), and more extensive necrosis involving the pancreatic body and tail (both *p* < 0.05), anatomical sites where necrosis is more prone to causing SVT. These findings indicate preferential AC use in patients with greater local complications and consequently more severe disease. Hospital stay was also longer in the AC group (20 vs. 15 days, *p* = 0.012; Table 2).

### 3.2. Splanchnic Vein Thrombosis and Anticoagulation

The splenic vein was the most commonly involved vessel (78.7%), followed by the portal vein (62.4%) and superior mesenteric vein (59.7%). Multi-vessel involvement (≥2 veins) occurred in 65.5% of patients. Concomitant DVT was present in 14% and PE in 1.6%, both significantly more frequent in the AC group (*p* < 0.05), reflecting selective AC use for more extensive thrombotic disease (Table 3).

Eighty-nine patients (34.5%) received AC. Among these, AC was administered for splenic vein thrombosis in 80.9%, portal vein thrombosis in 67.4%, and superior mesenteric vein thrombosis in 62.9%. LMWH was the most common regimen (*n* = 73, 82.0%), followed by DOACs (*n* = 12, 13.4%). LMWH regimens included once-daily subcutaneous dosing (*n* = 58), twice-daily dosing (*n* = 4), and dose adjustments based on vascular surgery consultations (*n* = 11). Treatment modifications occurred in 15 patients: 4 transitioned from LMWH to DOACs, 11 required dose adjustment based on vascular surgery consultation. Median AC duration was 42 days (range 1–83) (Table 4 and Table S1).

### 3.3. Recanalization Outcomes

At discharge, SVT recanalization was observed in 42 patients (16.3%). No significant difference was found between the AC and non-AC groups (15.7% vs. 16.6%, *p* = 0.862). After adjusting for baseline disease severity using mCTSI, AC remained not significantly associated with SVT recanalization (adjusted OR 0.92, 95% CI 0.46–1.87, *p* = 0.825; Table 3). Of note, mCTSI integrates multiple aspects of disease severity, including acute necrotic collections and pancreatic necrosis location, which were the main baseline imbalances between groups (Table 2). Site-specific analysis showed no significant difference for any venous segment: splenic vein (11.2% vs. 8.9%, *p* = 0.542), portal vein (9.0% vs. 5.9%, *p* = 0.357), or SMV (5.6% vs. 6.5%, *p* = 0.778). Deep vein thrombosis recanalization was observed in 9 patients, all in the AC group (10.0% vs. 0.0%, Table 3). As deep vein thrombosis testing were performed only when clinically indicated and deep vein thrombosis was itself an indication for AC, this finding is descriptive and is insufficient to establish evidence of AC efficacy. To explore whether recanalization differed by anticoagulant class, we separately analyzed patients receiving LMWH (*n* = 73) and DOACs (*n* = 12). Recanalization occurred in 11/73 (15.1%) of LMWH recipients vs. 3/12 (25%) of DOAC recipients. Given the small number of DOAC recipients, this analysis is exploratory and no statistical comparison was performed.

Among the anticoagulated patients, those with SVT recanalization (*n* = 14) had lower rates of smoking (7.1% vs. 42.7%, *p* = 0.012) and alcohol use (21.4% vs. 46.7%, *p* = 0.080), shorter prothrombin time (12.2 vs. 13.5 s, *p* = 0.038) and lower INR (1.07 vs. 1.15, *p* = 0.049), as well as a higher rate of necrosectomy (78.6% vs. 53.3%, *p* = 0.080) compared to those without recanalization (Table 5). Although the differences in alcohol use and necrosectomy did not reach statistical significance, likely due to the limited number of recanalization events, the consistent pattern across lifestyle, coagulation, and source control suggests that local factors may play a key role in thrombus resolution. To explore whether recanalization was attributable to surgical debridement or to AC itself, we stratified patients by necrosectomy status. Among the patients who underwent necrosectomy, recanalization occurred in 11/51 (21.6%) of those receiving AC vs. 21/83 (25.3%) of those not receiving AC. Among the patients who did not undergo necrosectomy, recanalization occurred in 3/38 (7.9%) vs. 7/86 (8.1%). Given the small number of events, these analyses are exploratory.

In a descriptive sensitivity analysis restricted to patients whose follow-up CECT was performed at approximately 30 days after SVT diagnosis (*n* = 29; 11 in the AC group and 18 in the non-AC group), recanalization occurred in 2/11 (18.2%) of the AC group vs. 4/18 (22.2%) of the non-AC group, and bleeding occurred in 3/11 (27.3%) vs. 2/18 (11.1%). Given the very small sample size, no statistical comparison was performed, and these data are presented descriptively only.

### 3.4. Bleeding Complications

Bleeding complications occurred in 40 patients (15.5%). The AC group had a significantly higher incidence of bleeding compared to the non-AC group (27.0% vs. 9.5%, *p* < 0.001) (Table 3). Major bleeding did not differ significantly between groups (4.3% vs. 1.2%, *p* = 0.214). The excess bleeding risk was driven predominantly by clinically significant hemoglobin decline (7.9% vs. 1.2%, *p* = 0.015), a CRNMB endpoint. After adjusting for baseline mCTSI imbalance (Table 2), AC remained significantly associated with bleeding (multivariate logistic regression: OR 3.20, 95% CI 1.58–6.46, *p* = 0.001; Table 3). To explore whether bleeding differed by anticoagulant class, we separately analyzed patients receiving LMWH (*n* = 73) and DOACs (*n* = 12). Bleeding complications occurred in 23/73 (31.5%) of LMWH recipients vs. 1/12 (8.3%) of DOAC recipients. Given the small number of DOAC recipients, this analysis is exploratory and no statistical comparison was performed.

Specific bleeding events in the AC vs. non-AC groups included: major bleeding (4.3% vs. 1.2%, *p* = 0.214) and CRNMB, including intra-abdominal hemorrhage (10.1% vs. 4.7%), gastrointestinal hemorrhage (5.6% vs. 3.6%), and clinically significant hemoglobin decline (7.9% vs. 1.2%, *p* = 0.015). Occult blood positivity was also higher in the AC group in both stool (3.4% vs. 0.6%) and urine (2.2% vs. 0%), though these differences were not statistically significant. Among patients with AC-associated major bleeding, four required endoscopic hemostasis (all achieved with titanium clip placement, one with adjunctive electrocoagulation). Two patients in the non-AC group underwent splenectomy with pancreatic tail resection for abdominal bleeding.

### 3.5. Mortality

Two in-hospital deaths due to multiorgan failure occurred (one in the AC group, one in the non-AC group). During 90-day follow-up, two additional deaths occurred, both in the AC group (one from intra-abdominal hemorrhage, one from multiorgan failure), resulting in a total of three deaths in the AC group and one in the non-AC group (Table 3).

## 4. Discussion

### 4.1. Summary of Main Findings

This prospective cohort study, the largest to date (*n* = 258), demonstrates three key findings. First, AC was not associated with improved short-term SVT recanalization (15.7% vs. 16.6%, *p* = 0.862). Second, AC was associated with a higher incidence of in-hospital bleeding complications (27.0% vs. 9.5%, *p* < 0.001), driven by clinically significant hemoglobin decline. Third, among the anticoagulated patients, recanalization had a numerically higher rate of necrosectomy (78.6% vs. 53.3%, *p* = 0.080), a hypothesis-generating observation that warrants further investigation.

### 4.2. SVT Characteristics in Our Cohort

The overall incidence of SVT in our cohort was 23.7%, with the splenic vein most commonly affected, followed by the portal vein and superior mesenteric vein. These patterns align with certain prior reports [28,29]. Notably, we also identified concomitant extra-splanchnic thrombotic complications, including DVT in 14.0% and PE in 1.6% of patients, confirming that moderate-severe AP predisposes to systemic thrombotic events [30]. Patients with SVT had a significantly prolonged hospitalization compared to those without, underscoring the clinical burden of this vascular complication [6].

### 4.3. Anticoagulation Practices

In our cohort, AC was administered to 89 patients (34.5%), with LMWH as the most common regimen (82.0%). Among the anticoagulated patients, splenic vein thrombosis was present in 80.9%, portal vein thrombosis in 67.4%, and SMV thrombosis in 62.9%, with 65.5% of cases involving two or more vessels. AC regimens (LMWH or DOACs) were comparable to previous literature, and selective AC policies align with recommendations against universal AC due to frequent AP-related interventions [17]. Unlike prior studies, no patients received warfarin [20,21]; DOACs were increasingly used for concomitant DVT [23]. The anticoagulant regimens in our cohort were heterogeneous, with LMWH used in 82.0% and DOACs in 13.4% of treated patients. Although the small number of DOAC recipients precludes a definitive comparison, recanalization rates appeared similar between the LMWH and DOAC recipients, whereas bleeding events occurred predominantly during LMWH therapy (23/24). These findings are consistent with the known pharmacokinetic differences between these agents and support the need for standardized regimens in future trials.

The median AC duration in our cohort was 42 days, which is shorter than the recommended minimum of 3 months for provoked SVT [31,32] but comparable to the median time to recanalization reported in some studies (e.g., 30 days) [17]. Despite this adequate duration, no recanalization benefit was observed, suggesting that factors other than treatment duration may explain the lack of AC efficacy. The AC practice at our center is consistent with these reports (Table 4).

### 4.4. Why Does AC Fail to Improve SVT Recanalization?

In our cohort, the in-hospital SVT recanalization rate was only 16.3%, with no significant difference between AC and non-AC groups (15.7% vs. 16.6%, *p* = 0.862). Although the AC group had more severe disease at the baseline, reflecting preferential AC use in higher-risk patients, multivariable adjustment for measurable confounders did not materially change the null finding for recanalization (adjusted OR 0.92, *p* = 0.825). This adjusted estimate should be interpreted as an association after accounting for disease severity, rather than as the independent treatment effect of AC, given that residual confounding cannot be excluded. Despite this limitation, this null finding aligns with the meta-analysis by Sissingh et al. (2022) [20] and the recent French cohort study by Maire et al. [15], but differs from Yin et al. (2023), who reported a significant recanalization benefit (RR 1.69) [19]. The lower recanalization rate in our cohort may reflect our focus on in-hospital rather than long-term outcomes, as some retrospective studies with longer follow-up have reported higher rates (e.g., 54.5% with AC at 2 years [16]).

Thrombus location also matters: AC shows efficacy for portal vein thrombosis (66.7% recanalization) but not for isolated splenic vein thrombosis (18.2%) [17], which is consistent with our observation that AC significantly improved DVT recanalization (10.0% vs. 0.0%, *p* < 0.001) but not SVT. However, because deep vein thrombosis testing was clinically driven and deep vein thrombosis itself prompted AC, this observation is exploratory and is insufficient to establish evidence of a differential treatment effect. It is nonetheless consistent with the hypothesis that deep vein thrombosis and SVT in AP have distinct mechanisms: deep vein thrombosis in AP is primarily driven by systemic hypercoagulability and immobilization, conditions that respond to systemic AC [33], whereas SVT is predominantly driven by local inflammation and mechanical compression from pancreatic necrosis [6,28], mechanisms not reversed by short-term AC [28]. Supporting this, among the anticoagulated patients, those who achieved recanalization had higher rates of acute necrotic collections and more extensive pancreatic necrosis [6,16,18], as well as a higher rate of necrosectomy (78.6% vs. 53.3%, Table 5), suggesting that source control and effective debridement of necrotic material may create favorable conditions for thrombus resolution. To address the potential confounding effect of surgical debridement, we stratified patients by necrosectomy status. Although limited by small event numbers, these analyses suggest that necrosectomy may be associated with recanalization independent of AC, supporting further investigation of source control as a potential determinant of thrombus resolution. Given the limited number of events, these subgroup analyses are exploratory and hypothesis-generating and require validation in larger studies. Nearly half of our patients underwent pancreatic necrosectomy (51.9%), reflecting our institution’s role as a specialized surgical referral center [34].

Another important methodological consideration is the differential observation window between groups. The AC group had a significantly longer hospital stay (20 vs. 15 days, *p* = 0.012), and correspondingly, a longer interval from SVT diagnosis to follow-up CECT. This differential observation window complicates the comparison of recanalization between groups. A time-to-event analysis would have been preferable but was not feasible, as the exact timing of recanalization was not recorded. A descriptive sensitivity analysis restricted to patients with follow-up CECT at approximately 30 days after SVT diagnosis showed a similar pattern to the primary analysis, although the sample size was too small for statistical inference. This analysis is exploratory and does not resolve the unequal observation period, which remains a limitation of this study.

### 4.5. Bleeding Risk: A Major Clinical Concern

The 27.0% in-hospital bleeding rate with AC in our cohort is substantially higher than most retrospective reports [17] but consistent with recent studies showing increased bleeding with AC. For instance, Jajoo et al. reported 50% clinically significant bleeding requiring treatment discontinuation [14], and Norton et al. reported 16% bleeding in the AC group vs. 5% in the non-AC group [23]. This consistency across studies suggests that retrospective studies may have systematically underestimated AC-associated bleeding complications in AP. Notably, CRNMB accounted for the majority of excess bleeding in our cohort (intra-abdominal hemorrhage: 10.1% vs. 4.7%; clinically significant hemoglobin decline: 7.9% vs. 1.2%). In total, AC was associated with a significantly higher incidence of bleeding complications, driven predominantly by clinically significant hemoglobin decline (7.9% vs. 1.2%, *p* = 0.015) rather than major bleeding (4.3% vs. 1.2%, *p* = 0.214). As treatment was not randomized, this association cannot be interpreted causally.

This differential pattern of bleeding events may explain the apparent discrepancy between our findings and previous meta-analyses that reported no significant increase in bleeding with AC [19,20]. In many prior studies, bleeding outcomes were often not defined using standardized criteria, making it difficult to ascertain whether non-major but clinically relevant events were systematically captured or reported. The hemoglobin decline observed in our cohort may prompt diagnostic workup, transfusion, or temporary AC interruption, even if it does not lead to permanent discontinuation. Prior meta-analyses may have systematically underestimated the full spectrum of AC-associated bleeding due to the lack of standardized bleeding definitions and the inherent limitations of retrospective data collection, where CRNMB events are more likely to be underreported [23]. Our findings are further corroborated by a meta-analysis: Yin et al. reported a recanalization benefit (RR 1.69) but only a trend toward increased bleeding (RR 1.98) [19]. However, this meta-analysis did not include the recently published prospective studies (e.g., Jajoo et al. [14] and our cohort), which have consistently demonstrated significantly increased bleeding risk with AC, potentially explaining the neutral bleeding finding in prior meta-analyses.

Importantly, as treatment was not randomized, we cannot exclude the possibility that some bleeding events were related to the underlying pancreatitis rather than to AC itself. However, in patients with hemorrhagic pancreatitis and concurrent SVT, AC was not associated with clinically significant bleeding [35], and Bandi et al. demonstrated that SVT alone did not significantly increase procedure-related bleeding [36]. These findings may suggest that the excess bleeding in our cohort is more likely attributable to AC than to an inherent vulnerability of SVT-affected vessels, although residual confounding cannot be excluded.

### 4.6. Clinical Implications

Although our findings should be regarded as hypothesis-generating given the observational design, they suggest that routine in-hospital AC for AP-associated SVT should be approached with caution. The lack of association with short-term recanalization, together with the higher incidence of bleeding complications, argues against a default AC strategy. Source control may warrant further investigation as a potential contributor to thrombus resolution, supporting the prioritization of interventional drainage over systemic AC in suitable candidates. These hypotheses require confirmation in randomized controlled trials.

### 4.7. Limitations

Several limitations merit acknowledgment. First, the in-hospital follow-up may underestimate late recanalization events, though this was by design to answer the clinically relevant question about early benefit during hospitalization. Of note, the median AC duration in our cohort was comparable to that reported in other studies. Second, this is a single-center study conducted at a high-volume referral center, which may limit generalizability; however, consecutive enrollment and independent imaging review may minimize selection bias. Third, AC decisions were based on clinician judgment rather than randomization. The AC group had more severe disease at the baseline, introducing indication bias. Although we adjusted for baseline disease severity using mCTSI (which integrates acute necrotic collections and pancreatic necrosis location), residual confounding from unmeasured variables cannot be excluded. The anatomical distribution of SVT and the number of affected vessels did not differ significantly between groups. Concomitant DVT/PE was more frequent in the AC group; however, DVT/PE screening was performed only when clinically indicated and was not part of the original study design, so this difference may partly reflect differential detection. Because of these considerations, and given the sample size, a more comprehensive multivariable model incorporating all variables relevant to treatment allocation or propensity score analysis was not feasible. As treatment was not randomized, the association between AC and bleeding cannot be interpreted causally, and we cannot definitively exclude the possibility that some bleeding events were related to the underlying pancreatitis. These uncertainties underscore the need for rigorously designed randomized trials to evaluate the role of AC in AP-associated SVT. Fourth, recanalization was assessed on the last CECT before discharge, and the AC group had a longer interval from SVT diagnosis to follow-up CECT. This differential observation window complicates the comparison between groups. A time-to-event analysis was not feasible, as the exact timing of recanalization was not recorded. A descriptive sensitivity analysis restricted to patients with follow-up at approximately 30 days showed a similar pattern but was too small for statistical inference. Future studies with serial imaging at predefined intervals are needed. Fifth, no formal sample size calculation was performed, and the limited number of SVT recanalization events reduced statistical power for multivariable adjustment and subgroup analyses; accordingly, the exploratory findings regarding necrosectomy should be interpreted with caution and require validation in larger studies. Sixth, the lack of a standardized AC protocol and the heterogeneity of regimens (LMWH vs. DOACs, variable dosing schedules, and treatment modifications) reflect real-world clinical practice; this heterogeneity may have contributed to variability in treatment exposure and represents a limitation of this observational study.

## 5. Conclusions

Existing evidence on AC for AP-associated SVT is predominantly retrospective and conflicting. In this observational cohort, in-hospital AC was not associated with improved short-term SVT recanalization but was associated with a higher incidence of bleeding complications, driven predominantly by clinically significant hemoglobin decline. These observations require confirmation in randomized trials to determine whether AC provides a net benefit in this population.

## Figures and Tables

**Table 1:** Summary of key studies on anticoagulation for AP-associated SVT.

Author, Year	Country	Study Design	N (AC/Non-AC)	Key Findings on Recanalization	Key Findings on Bleeding
Original Studies					
Jajoo, 2025 [14]	USA	Retrospective	43 (19/23)	No report	↑ bleeding (50% vs. 0%)
Maire, 2025 [15]	France	Retrospective	88 (69/19)	No difference (41% vs. 47%)	No difference (14% vs. 10%)
Oyon, 2023 [16]	Spain	Post hoc analysis	46 (33/13)	No difference (54.5% vs. 30.8%)	One fatal thrombotic complication on LMWH
Thejasvin, 2022 [17]	UK	Retrospective	109 (74/35)	PV with AC: 66.7%, SpV with AC: 18.2%; non-AC: 31.4%	No difference (6.8% vs. 0%)
Pagliari, 2020 [8]	Italy	Retrospective	27 (16/11)	↑ (68.7% vs. 27.3%, *p* = 0.03)	No bleeding in either group
Junare, 2020 [18]	India	Prospective	24 (12/12)	No difference (50% vs. 41.7%)	No difference (25% vs. 0.0%)
Zhou, 2020 [7]	China	Retrospective	273 (pooled)	AC ↓ SVT incidence (21.3% vs. 46.2%, *p* < 0.001)	No difference (19.2% vs. 14.8%)
Meta-analyses					
Yin, 2023 [19]	China	Meta-analysis	698 (pooled)	↑ recanalization (RR 1.69; 95% CI 1.29–2.19)	Trend ↑ bleeding (RR 1.98; 95% CI 0.93–4.22)
Sissingh, 2022 [20]	Netherlands	Meta-analysis	233 (109/124)	No difference (ARD 9%; 95% CI −0.11 to 0.28)	No difference (ARD 3%; 95% CI −0.06 to 0.12)
Chandan, 2021 [9]	USA	Meta-analysis	239 (103/136)	↑ recanalization (RR 1.6; 95% CI 0.98–3.88)	No difference (RR 1.95; 95% CI 0.97–3.92)
Anis, 2021 [21]	UK	Meta-analysis	943 (264/679)	↑ recanalization (OR 0.51; 95% CI 0.31–0.83)	No difference (OR 2.27; 95% CI 0.81–6.37)
Valeriani, 2021 [22]	Italy	Meta-analysis	7969 (pooled)	↑ recanalization (RR 2.39; 95% CI 1.66–3.44)	↓ major bleeding (RR 0.73, 95% CI 0.58–0.92)
Norton, 2020 [23]	UK	Systematic review	198 (92/106)	AC: 14% vs. non-AC: 11%	AC: 16% vs. non-AC: 5%

Arrows indicate the direction of the reported effect: ↑, increased; ↓, decreased. AP: acute pancreatitis; SVT: splanchnic vein thrombosis; AC: anticoagulation; non-AC: without anticoagulation; LMWH: low molecular weight heparin; PV: portal vein; SpV: splenic vein; OR: odds ratio; RR: risk ratio; CI: confidence interval; ARD: absolute risk difference.

**Table 2:** Comparison of baseline characteristics and clinical outcome between non-AC and AC groups in SVT patients.

Parameters	SVT Patients (*n* = 258)	Non-AC Group (*n* = 169)	AC Group (*n* = 89)	*p* Value
Demographics				
Age, years, median (IQR)	47 (38, 57)	47 (37, 57)	47 (39, 58)	0.614
Gender, male, %	177 (68.6)	118 (69.8)	59 (66.3)	0.561
Body mass index, kg/m^2^ median (IQR)	22.0 (19.7, 24.4)	22.0 (19.5, 24.1)	22.7 (20.8, 25.0)	0.070
Charlson comorbidity index, median (IQR)	1 (0, 3)	1 (0, 3)	3 (0, 3)	0.478
Smoking, %	91 (35.3)	58 (34.3)	33 (37.1)	0.659
Alcohol, %	101 (39.1)	63 (37.3)	38 (42.7)	0.397
AP episode				
First, %	180 (69.8)	115 (68.0)	65 (73.0)	0.127
Second, %	33 (12.8)	19 (11.2)	14 (15.7)	
Third or more, %	45 (17.4)	35 (20.7)	10 (11.2)	
Etiology				
Biliary, %	83 (32.2)	51 (30.2)	32 (36.0)	0.362
Hypertriglyceridemia, %	119 (46.1)	78 (46.2)	41 (46.1)	
Alcoholics, %	11 (4.3)	6 (3.6)	5 (5.6)	
Others, %	45 (17.4)	34 (20.1)	11 (12.4)	
Coagulation Parameters on Admission				
Platelet, ×10^9^/L, median (IQR)	301 (223.5, 452.5)	340.5 (245.3, 486.3)	292 (222.5, 380.3)	0.921
Prothrombin Time, second, median (IQR)	13.1 (12.3, 13.9)	13 (12.4, 13.9)	13.2 (12.1, 15.2)	0.527
APTT, second, median (IQR)	28.8 (27.2, 31.2)	28.4 (26.9, 30.9)	29.0 (27.1, 31.8)	0.153
INR, median (IQR)	1.14 (1.08, 1.23)	1.15 (1.09, 1.23)	1.14 (1.07, 1.24)	0.690
D-Dimer, mg/L, median (IQR)	4.32 (2.63, 9.31)	4.32 (2.82, 9.51)	4.89 (2.79, 10.48)	0.335
Fibrinogen, g/L, median (IQR)	3.88 (2.93, 4.74)	3.91 (3.02, 4.72)	4.00 (2.89, 4.93)	0.819
Fibrin Degradation Products, mg/L, median (IQR)	10.5 (6.4, 18.3)	10.5 (6.3, 18.4)	10.2 (6.7, 17.4)	0.989
Antithrombin III, %, median (IQR)	79.7 (69.0, 92.4)	79.5 (69.0, 92.0)	79.1 (69.0, 95.8)	0.724
mCTSI, median (IQR)	8 (8, 10)	8 (8, 10)	8 (8, 10)	0.023
Local complication				
Acute peripancreatic fluid collection, %	196 (76.0)	130 (76.9)	66 (74.2)	0.621
Pseudocyst, %	29 (11.2)	22 (13.0)	7 (7.9)	0.213
Acute necrotic collections, %	99 (38.4)	57 (33.7)	42 (47.2)	0.035
Location of necrosis				
Head, %	88 (34.1)	56 (33.1)	32 (36.0)	0.650
Neck, %	83 (32.2)	52 (30.8)	31 (34.8)	0.507
Body, %	198 (76.7)	122 (72.2)	76 (85.4)	0.017
Tail, %	204 (79.1)	126 (74.6)	78 (87.6)	0.014
Combined, %	70 (27.1)	40 (23.7)	30 (33.7)	0.085
Extent of necrosis				
<30%	80 (31.0)	59 (34.9)	21 (23.6)	0.062
30–50%	112 (43.4)	69 (40.8)	43 (48.3)	0.249
≥50%	58 (22.5)	34 (20.1)	24 (27.0)	0.210
Necrosectomy, %	134 (51.9)	83 (49.1)	51 (57.3)	0.211
Hospital LOS, median (IQR)	17 (10, 28)	15 (9, 26)	20 (11, 37)	0.012

Non-AC: without anticoagulation; AC: anticoagulation; SVT: splanchnic vein thrombosis; IQR: interquartile range; AP: acute pancreatitis; APTT: activated partial thromboplastin time; INR: international normalized ratio; mCTSI: modified computed tomography severity index; LOS: length of stay.

**Table 3:** Comparison of splanchnic vein thrombosis and related complications between non-AC and AC groups in SVT patients.

Parameters	SVT Patients (*n* = 258)	Non-AC Group (*n* = 169)	AC Group (*n* = 89)	*p* Value
Type of vein				
Splenic vein, %	203 (78.7)	131 (77.5)	72 (80.9)	0.528
Portal vein, %	161 (62.4)	101 (59.8)	60 (67.4)	0.228
Superior mesenteric vein, %	154 (59.7)	98 (58.0)	56 (62.9)	0.443
SpV + PV, %	76 (29.5)	46 (27.2)	30 (33.7)	0.277
SpV + SMV, %	79 (30.6)	48 (28.4)	31 (34.8)	0.287
PV + SMV, %	72 (27.9)	43 (25.4)	29 (32.6)	0.224
SpV + PV + SMV, %	104 (40.3)	66 (39.1)	38 (42.7)	0.571
Concomitant venous thromboembolism				
Pulmonary embolism, %	4 (1.6)	0 (0.0)	4 (4.5)	0.025
Deep vein thrombosis, %	36 (14.0)	6 (3.6)	30 (33.3)	<0.001
Number of affected vessels				
One, %	78 (30.2)	54 (32.7)	24 (25.8)	0.575
Two or three, %	169 (65.5)	107 (63.3)	62 (69.7)	
Recanalization				
SVT, %	42 (16.3)	28 (16.6)	14 (15.7)	0.862 ^a^
Splenic vein, %	25 (9.7)	15 (8.9)	10 (11.2)	0.542
Portal vein, %	18 (7.0)	10 (5.9)	8 (9.0)	0.357
Superior mesenteric vein, %	16 (6.2)	11 (6.5)	5 (5.6)	0.778
Deep vein thrombosis, %	9 (3.5)	0 (0.0)	9 (10.0)	<0.001
Complications of SVT ^b^				
Gastroesophageal varices, %	72 (27.9)	47 (27.8)	25 (28.1)	0.962
Collateral formation, %	45 (17.4)	24 (14.5)	21 (22.6)	0.059
Splenic infarction, %	12 (4.7)	7 (4.1)	5 (5.6)	0.823
Portal cavernoma, %	6 (2.3)	3 (1.8)	3 (3.2)	0.709
Portal hypertension	146 (56.6)	94 (55.6)	52 (58.5)	0.666
Ascites	152 (58.9)	100 (59.5)	52 (57.8)	0.786
Bleeding complications	40 (15.5)	16 (9.5)	24 (27.0) ^c^	<0.001 ^d^
Major Bleeding	6 (2.3)	2 (1.2) ^e^	4 (4.3) ^f^	0.214
CRNMB				
Intra-abdominal hemorrhage, %	17 (6.6)	8 (4.7)	9 (10.1)	0.098
Gastrointestinal hemorrhage, %	11 (4.3)	6 (3.6)	5 (5.6)	0.647
Clinically significant hemoglobin decline, %	9 (3.5)	2 (1.2)	7 (7.9)	0.015
Minor Bleeding				
Stool positive for occult blood, %	4 (1.6)	1 (0.6)	3 (3.4)	0.235
Urine positive for occult blood, %	2 (0.8)	0 (0)	2 (2.2)	0.226
Other ^g^	4 (1.6)	1 (0.6)	3 (3.4)	0.235
90 day follow-up mortality, %	4 (1.6)	1 (0.6)	3 (3.3)	0.243

Non-AC: without anticoagulation; AC: anticoagulation; SVT: splanchnic vein thrombosis; SpV: splenic vein; PV: portal vein; SMV: superior mesenteric vein; CRNMB: clinically relevant non-major bleeding; ^a^ After adjusting for baseline mCTSI imbalance (Table 2), AC remained not significantly associated with SVT recanalization: OR 0.92, 95% CI 0.46–1.87, *p* = 0.825. ^b^ No patients had liver failure, small bowel ischemia, and necrosis. ^c^ Of the 24 bleeding events in the AC group, 23 occurred during LMWH therapy and only 1 during DOAC therapy. ^d^ After adjusting for baseline mCTSI imbalance (Table 2), AC remained significantly associated with bleeding risk: OR 3.20, 95% CI 1.58–6.46, *p* = 0.001. ^e^ Two patients in the non-AC group underwent combination of splenectomy with the resection of the pancreatic tail due to abdominal bleeding. ^f^ Endoscopy intervention was performed in four patients with gastrointestinal hemorrhage, all of whom achieved hemostasis with titanium clip placement, including one case requiring adjunctive electrocoagulation. ^g^ Percutaneous transhepatic cholangial drainage yielded bloody fluid, and CT revealed abdominal hematoma formation and hemorrhagic peripancreatic walled-off necrosis.

**Table 4:** The anticoagulation regime in the AC group.

AC Regimens		Patient	AC Start Time			Duration		
			<7 d	7–14 d	>14 d	<7 d	7–14 d	>14 d
LMWH	qd	58	40	9	9	22 ^a^	22	14
	q12h	4	4			1	3	
	qd-q12h	5	2	1	2		2	3
	q12h-qd	6	3	2				5
DOACs		12	5	4	2	3	8	1
Transitioning from LMWH to DOACs		4	2		2	1	1	2

AC: anticoagulation; LMWH: low molecular weight heparin; DOACs: direct oral anticoagulants; ^a^ Five cases were discontinued AC due to bleeding complication.

**Table 5:** Comparison of baseline characteristics, AC and clinical outcomes in the non-recanalization and recanalization groups.

Parameters	Non-Recanalization (*n* = 75)	Recanalization (*n* = 14)	*p* Value
Baseline characteristics			
Age, years, median (IQR)	48 (40, 58)	52 (48, 58)	0.420
Gender, male, %	51 (68.0)	8 (57.1)	0.631
Body mass index, kg/m^2^ median (IQR)	22.9 (21.0, 25.0)	21.2 (19.5, 24.0)	0.236
Charlson comorbidity index, median (IQR)	1.0 (0.0, 3.0)	1 (0.0, 3.0)	0.577
Smoking, %	32 (42.7)	1 (7.1)	0.012
Alcohol, %	35 (46.7)	3 (21.4)	0.080
Acute pancreatitis episode			
First, %	54 (72.0)	11 (78.6)	0.844
Second, %	12 (16.0)	2 (14.3)	
Third or more, %	9 (12.0)	1 (7.1)	
Etiology			
Biliary	25 (33.3)	7 (50.0)	0.516
Hypertriglyceridemia	36 (48.0)	5 (35.7)	
Alcoholics	5 (6.7)	0 (0.0)	
Others	9 (12.0)	2 (14.3)	
Coagulation Parameters on Admission			
Platelet, ×10^9^/L, median (IQR)	284 (216, 389)	328 (221, 387)	0.706
Prothrombin Time, second, median (IQR)	13.5 (12.4, 15.5)	12.2 (12.0, 12.5)	0.038
APTT, second, median (IQR)	29.0 (27.1, 32.2)	30.0 (27.7, 32.0)	0.581
INR, median (IQR)	1.15 (1.08, 1.26)	1.07 (1.05, 1.20)	0.049
D-Dimer, mg/L, median (IQR)	4.70 (2.64, 10.68)	4.59 (3.38, 9.01)	0.989
Fibrinogen, g/L, median (IQR)	3.87 (2.89, 4.74)	3.74 (2.28, 5.22)	0.559
Fibrin Degradation Products, mg/L, median (IQR)	10.20 (6.55, 18.60)	10.60 (7.00, 15.50)	0.850
Antithrombin III, %, median (IQR)	79.6 (66.0, 95.7)	79.1 (71.4, 96.3)	0.961
mCTSI, median (IQR)	8 (8, 10)	8 (8, 10)	0.357
Splanchnic Vein Thrombosis			
Type of vein			
Splenic vein, %	59 (78.7)	13 (92.9)	0.385
Portal vein, %	51 (68.0)	9 (64.3)	1.000
Superior mesenteric vein, %	48 (64.0)	8 (57.1)	0.626
SpV + PV, %	24 (32.0)	6 (42.9)	0.631
SpV + SMV, %	25 (33.3)	6 (42.9)	0.703
PV + SMV, %	26 (34.7)	3 (21.4)	0.510
SpV + PV + SMV, %	33 (44.0)	5 (35.7)	0.565
Concomitant venous thromboembolism			
Pulmonary embolism, %	3 (4.0)	1 (7.1)	1.000
Deep vein thrombosis, %	16 (21.3)	1 (7.1)	0.385
Number of affected vessels			
One, %	21 (28.0)	3 (21.4)	0.626
Two or three, %	51 (68.0)	11 (78.6)	
Clinical Outcomes			
Local complication			
Acute peripancreatic fluid collection, %	56 (74.7)	10 (71.4)	1.000
Pseudocyst, %	5 (6.7)	2 (14.3)	0.666
Acute necrotic collections, %	34 (45.3)	8 (57.1)	0.416
Location of necrosis			
Body, %	65 (86.7)	11 (78.6)	0.708
Head, %	27 (36.0)	5 (35.7)	0.984
Neck, %	28 (37.3)	3 (21.4)	0.400
Tail, %	66 (88.0)	12 (85.7)	1.000
Combined, %	26 (36.7)	4 (28.6)	0.893
Extent of necrosis			
<30%, %	15 (20.0)	6 (42.9)	0.132
30–50%, %	38 (50.7)	5 (35.7)	0.304
<50%, %	21 (28.0)	3 (21.4)	0.857
Necrosectomy, %	40 (53.3)	11 (78.6)	0.080
Mortality, %	1 (1.3)	0 (0.0)	1.000
Hospital LOS, median (IQR)	20 (11, 33)	25 (9, 45)	0.656

These analyses are exploratory due to the limited number of recanalization events. AC: anticoagulation; IQR: interquartile range; APTT: activated partial thromboplastin time; INR, international normalized ratio; mCTSI: modified computed tomography severity index; SpV: splenic vein; PV: portal vein; SMV: superior mesenteric vein; LOS: Length of Stay.

## Data Availability

The datasets generated and analyzed during the current study are available from the corresponding authors on reasonable request.

## Supplementary Materials

The following supporting information can be downloaded at: https://www.mdpi.com/article/10.3390/jcm15207824/s1, Table S1a. Anticoagulation regimens, modifications, and discontinuation (*n* = 89). Table S1b. Timing, interventions, and outcomes (*n* = 89).
