# Supplementary material for: Anticoagulation for Splanchnic Vein Thrombosis in Moderate-Severe Acute Pancreatitis: In-Hospital Outcomes from a Prospective Cohort Study
Source: J Clin Med. 2026 Oct 10;15(20):7824. doi: 10.3390/jcm15207824 (PMC13705513; doi:10.3390/jcm15207824)
Supplement: Supplementary file 1 [file jcm-15-07824-s001.zip › jcm-4542754-supplementary.pdf]

Supplementary Table S1a. Anticoagulation regimens, modifications, and discontinuation (*n* = 89)

ID	mCTSI	AC indication	Drug	Frequency	Regimen change	Reason for change	AC stop	Reason for stop
1	10	PV+SMV	LMWH	qd	-	-	Yes	Other reason[*]
2	10	PV	LMWH	qd	-	-	Yes	Intra-abdominal hemorrhage
3	10	PV+SMV	LMWH	qd	-	-	No	-
4	10	PV+SMV	LMWH	qd	-	-	Yes	Intra-abdominal hemorrhage
5	10	PV	LMWH	qd	-	-	No	-
6	8	SpV progression	LMWH	qd	-	-	No	-
7	10	PV	LMWH	qd	-	-	Yes	Intra-abdominal hemorrhage
8	8	PV+SMV	LMWH	qd	-	-	-	-
9	10	PV+SMV	LMWH	qd	-	-	Yes	Intra-abdominal hemorrhage
10	8	SMV	LMWH	qd	-	-	No	-
11	8	SpV progression	LMWH	qd	-	-	No	-
12	8	PV+SMV	LMWH	qd	-	-	No	-
13	10	PV+SMV	LMWH	qd	-	-	No	Other reason[*]
14	8	SMV	LMWH	qd	-	-	Yes	Clinically significant hemoglobin decline
15	10	SMV	LMWH	qd	-	-	No	-
16	8	SMV	LMWH	qd	-	-	No	-
17	8	SpV progression	LMWH	qd	→ q12h	No change	Yes	Clinically significant hemoglobin decline
18	10	PV	LMWH	q12h	→ qd	Thrombus partial resolution	Yes	Clinically significant hemoglobin decline, Stool positive for occult blood
19	10	PV+SMV	LMWH	qd	-	-	No	-

20	6	SMV	LMWH	qd	-	-	No	-
21	8	PV	DOAC	qd	-	-	No	-
22	8	SpV progression	LMWH	q12h	→ qd	Thrombus partial resolution	Yes	Gastrointestinal hemorrhage
23	6	PV	LMWH	qd	-	-	No	-
24	8	SMV	LMWH	qd	→ q12h	Thrombus progression	Yes	Intra-abdominal hemorrhage
25	8	PV+SMV	LMWH	qd	-	-	Yes	Intra-abdominal hemorrhage
26	8	PV	DOAC	qd	-	-	No	-
27	8	SpV progression	LMWH	qd	→ q12h	No change	No	-
28	8	PV	DOAC	qd	-	-	No	-
29	8	SpV progression	LMWH	qd	-	-	No	-
30	10	PV+SMV	DOAC	q12h	-	-	No	-
31	8	SMV	LMWH	qd	-	-	No	-
32	10	PV+SMV	LMWH	qd	-	-	No	-
33	10	PV+SMV	LMWH	qd	→ DOAC	MDT adjustment	No	-
34	8	PV+SMV	LMWH	qd	-	-	No	-
35	8	PV+SMV	DOAC	qd	-	-	No	-
36	8	PV+SMV	LMWH	qd	-	-	No	-
37	10	SMV	LMWH	qd	-	-	No	-
38	8	PV+SMV	DOAC	qd	-	-	No	-
39	8	PV+SMV	LMWH	q12h	→ qd	Thrombus partial resolution	Yes	Gastrointestinal hemorrhage
40	8	SMV	LMWH	qd	-	-	No	-
41	8	SMV	LMWH	q12h	-	-	No	-
42	8	SpV progression	DOAC	qd	-	-	No	-
43	8	SpV progression	LMWH	qd	-	-	Yes	Gastrointestinal hemorrhage
44	10	PV+SMV	LMWH	qd	→ DOAC	MDT adjustment	No	-

45	10	SMV	LMWH	qd	-	-	No	-
46	10	PV	LMWH	qd	-	-	Yes	Gastrointestinal hemorrhage
47	8	SpV progression	LMWH	qd	-	-	No	-
48	8	SpV progression	LMWH	qd	-	-	No	-
49	8	PV+SMV	LMWH	qd	→ DOAC	MDT adjustment	No	-
50	10	PV	LMWH	qd	-	-	No	-
51	10	PV+SMV	LMWH	q12h	-	-	Yes	Clinically significant hemoglobin decline, Urine positive for occult blood
52	10	PV+SMV	LMWH	qd	-	-	No	-
53	8	SpV progression	LMWH	qd	-	-	Yes	Clinically significant hemoglobin decline, Stool positive for occult blood
54	10	PV	FPX	qd	-	-	No	-
55	6	SpV progression	DOAC	qd	-	-	No	-
56	10	PV+SMV	LMWH	qd	→ q12h	Thrombus progression	No	-
57	8	PV+SMV	LMWH	q12h	→ qd	Thrombus partial resolution	Yes	Intra-abdominal hemorrhage
58	6	PV+SMV	DOAC	qd	-	-	No	-
59	6	PV	LMWH	q12h	→ qd	Thrombus partial resolution	Yes	Gastrointestinal hemorrhage
60	6	PV+SMV	LMWH	qd	-	-	No	-
61	10	SpV progression	LMWH	qd	-	-	No	-
62	10	PV+SMV	LMWH	q12h	→ qd → DOAC	Thrombus partial resolution	No	-
63	10	PV+SMV	LMWH	qd	→ q12h	Thrombus progression	Yes	Intra-abdominal hemorrhage
64	6	SMV	LMWH	qd	-	-	No	-
65	8	SMV	LMWH	qd	-	-	No	-

66	10	SMV	LMWH	qd	-	-	Yes	Clinically significant hemoglobin decline, Stool positive for occult blood
67	8	PV	LMWH	qd	-	-	No	-
68	8	SpV progression	LMWH	qd	-	-	No	-
69	10	PV+SMV	LMWH	qd	-	-	No	-
70	10	SMV	LMWH	qd	-	-	No	-
71	10	PV	LMWH	qd	-	-	No	-
72	10	PV+SMV	LMWH	qd	-	-	No	-
73	10	PV+SMV	LMWH	q12h	-	-	Yes	Other reason*
74	8	PV+SMV	LMWH	qd	-	-	No	-
75	8	PV+SMV	LMWH	qd	-	-	No	-
76	10	PV+SMV	DOAC	qd	-	-	No	-
77	8	PV	LMWH	qd	-	-	No	-
78	10	PV+SMV	LMWH	q12h	-	-	No	-
79	8	SpV progression	LMWH	qd	-	-	Yes	Clinically significant hemoglobin decline
80	8	PV+SMV	LMWH	qd	-	-	No	-
81	8	PV+SMV	LMWH	q12h	-	-	No	-
82	8	PV+SMV	LMWH	qd	-	-	No	-
83	10	PV+SMV	DOAC	qd	-	-	Yes	Intra-abdominal hemorrhage, Urine positive for occult blood
84	6	SpV progression	LMWH	qd	-	-	No	-
85	10	PV+SMV	LMWH	qd	-	-	No	-

86	10	PV	LMWH	qd	-	-	No	-
87	8	PV+SMV	DOAC	qd	-	-	No	-
88	8	PV+SMV	LMWH	qd	-	-	No	-
89	10	PV+SMV	LMWH	qd	-	-	No	-

mCTSI, modified Computed Tomography Severity Index; AC, anticoagulation; PV, portal vein; SMV, superior mesenteric vein; LMWH, low molecular weight heparin; qd, once daily; SpV, splenic vein; DVT, deep vein thrombosis; q12h, every 12 hours; DOACs, direct oral anticoagulants; PE, pulmonary embolism; MDT, multidisciplinary team; FPX, Fondaparinux.

*Percutaneous transhepatic cholangial drainage yielded bloody fluid, and CT revealed abdominal hematoma formation and hemorrhagic peripancreatic walled-off necrosis.

Supplementary Table S1b. Timing, interventions, and outcomes (n = 89)

ID	AC start (day from SVT diagnosis)	Necrosectomy	Follow-up CECT (day from SVT diagnosis)	Recanalization	Bleeding
1	2	Yes	7	No	Yes
2	2	Yes	12	No	Yes
3	3	No	6	No	No
4	12	Yes	NA	No	Yes
5	5	Yes	10	No	No
6	4	No	7	No	No
7	0	No	34	No	Yes
8	0	No	14	No	No
9	2	Yes	49	No	Yes
10	0	No	7	No	No
11	3	No	8	No	No
12	0	No	59	No	No
13	6	Yes	54	No	Yes
14	3	Yes	10	No	Yes
15	2	Yes	23	No	No
16	2	No	11	No	No
17	0	No	33	No	Yes
18	2	No	26	No	Yes
19	1	Yes	6	Yes	No
20	1	No	12	No	No
21	3	Yes	37	Yes	No
22	1	Yes	46	No	Yes

23	2	Yes	NA	No	No
24	1	Yes	13	No	Yes
25	3	Yes	18	Yes	Yes
26	3	Yes	30	No	No
27	1	No	46	No	No
28	0	No	25	Yes	No
29	2	No	14	No	No
30	1	No	13	No	No
31	1	No	30	No	No
32	1	No	23	No	No
33	1	Yes	62	No	No
34	0	No	44	No	No
35	2	No	19	No	No
36	2	No	39	No	No
37	1	Yes	8	Yes	No
38	11	Yes	17	Yes	No
39	0	Yes	51	No	Yes
40	1	Yes	70	No	No
41	1	No	43	No	No
42	2	Yes	NA	No	No
43	3	No	19	No	Yes
44	3	Yes	38	No	No
45	3	Yes	22	Yes	No
46	6	No	26	No	Yes
47	1	No	47	No	No

48	3	No	18	No	No
49	3	Yes	60	No	No
50	2	No	10	No	No
51	2	No	30	No	Yes
52	1	No	44	No	No
53	1	No	21	No	Yes
54	1	No	18	No	No
55	1	Yes	9	No	No
56	5	Yes	7	Yes	No
57	8	Yes	6	No	Yes
58	0	No	9	No	No
59	1	Yes	18	Yes	Yes
60	2	Yes	NA	No	No
61	3	No	37	No	No
62	0	Yes	39	No	No
63	2	Yes	22	No	Yes
64	3	Yes	62	No	No
65	0	Yes	20	No	No
66	0	Yes	20	No	Yes
67	2	Yes	7	Yes	No
68	2	Yes	32	Yes	No
69	1	Yes	14	No	No
70	0	Yes	53	Yes	No
71	9	No	9	No	No
72	3	Yes	49	No	No

73	3	No	23	No	Yes
74	2	Yes	19	Yes	No
75	0	Yes	NA	No	No
76	7	Yes	NA	No	No
77	0	No	16	No	No
78	0	Yes	27	No	No
79	2	No	NA	Yes	Yes
80	8	Yes	21	No	No
81	1	Yes	10	No	No
82	0	Yes	20	No	No
83	2	No	10	No	Yes
84	3	Yes	3	No	No
85	1	No	41	No	No
86	4	Yes	42	No	No
87	1	Yes	NA	No	No
88	1	Yes	35	No	No
89	19	Yes	NA	No	No

AC, anticoagulation; SVT, splanchnic vein thrombosis; CECT, contrast-enhanced computed tomography; NA, not applicable (no follow-up CECT performed).
